# Challenge for the future: assessing the quality of abdominal surgery using composite quality measures

**DOI:** 10.1093/bjsopen/zrad116

**Published:** 2023-11-02

**Authors:** Giulia Capelli, Gaya Spolverato

**Affiliations:** Department of Surgical, Oncological and Gastroenterological Sciences (DiSCOG), University of Padua, Padua, Italy; Department of Surgery, ASST Bergamo Est, Seriate (Bergamo), Italy; Department of Surgical, Oncological and Gastroenterological Sciences (DiSCOG), University of Padua, Padua, Italy

Rajesh *et al*. propose a systematic review focusing on composite quality measures using administrative and clinical data as a tool to assess the overall quality of abdominal surgery^1^. The authors thoroughly analysed the included scores, providing detailed information on each outcome measure, analysing the methodology of development, and assessing strengths and weaknesses to examine their actual applicability in clinical practice. While acknowledging several limitations to the effectiveness of composite outcome measures as indicators of quality in abdominal surgery, the authors conclude that simpler scores—namely, ‘postoperative mortality rate, postoperative transfer to another hospital, postoperative length of stay’ (MTL), ‘hospital stay, readmission and mortality’ (HARM) and ‘days alive and out of hospital’ (DAOH)—should be further tested in large population datasets to implement their use as safety and quality tools.

Despite the well-known limitations of composite quality measures for assessing outcomes in abdominal surgery, efforts are underway to develop simple and reliable scores for use in clinical practice. It is now widely recognized that single measures do not capture the full patient experience and may be inadequate to assess the quality of care received. Moreover, some important but rare outcomes, such as mortality rate, are unreliable as quality measures because of their low event rate. As such, composite outcome measures that include a variety of outcomes should be considered as the way to go in the future to improve the quality of surgical care^2^.

While we agree with the authors that simpler scores should be easy and inexpensive to translate into everyday clinical practice, simplistic and arbitrarily selected outcomes do not represent the true outcome of surgery, which is strongly influenced by the patient's experience. Patient-reported outcome measures (PROMs) have gained increasing interest among researchers as a means to evaluate the quality of abdominal surgery. Although based on subjective experience, PROMs are accurately developed and rigorously validated, making them a reliable tool for assessing outcomes that are often underestimated by healthcare providers, such as pain and psychological distress^3^.

A word should also be spent on cancer patients requiring surgical treatment: in this particular case, the use of scores that only evaluate short-term outcomes fail to take into account some important factors that strongly influence patients’ prognosis. Some more specific scores have been proposed, including the Textbook Oncologic Outcomes (TOO), which includes both procedural and cancer-specific outcomes. Achievement of a TOO has been associated with improved long-term outcomes in several international studies, making it an outcome measure worthy of future research^4^.

Finally, a limitation of simpler composite quality measures is the inability to assign different weights to individual outcomes. Several interesting statistical methods are currently being tested that allow the clinical hierarchy to be taken into account within composite endpoints. For example, the Win Ratio (WR) analysis has been proposed to assign different weights to individual postoperative outcomes to identify patients who receive an overall benefit (that is ‘win’) from surgical treatment. The WR has recently been applied to Medicare beneficiaries undergoing hepatopancreatic surgery with interesting results and is currently being tested as a quality measure for various surgical oncology procedures^5^.

In conclusion, Rajesh *et al*. provide an interesting and consistent analysis of composite outcome measures available for clinical research. In the future, we should expect to see the development and validation of scores that are able to assign a hierarchy to postoperative outcomes, taking into account patient experience and the specific needs of oncologic patients. The real challenge will be to incorporate all these features into a score that is simple enough to be widely used in clinical practice.
